# Can Primary Cardiac Myxofibrosarcoma Grow Quickly from Zero to a Size Leading to Left-Sided Heart Failure within 9 Months?

**DOI:** 10.1155/2020/4241204

**Published:** 2020-12-08

**Authors:** Levan Karazanishvili, Eduard Limonjiani

**Affiliations:** ^1^EVEX Medical Corporation, Georgia; ^2^Tbilisi State Medical University, Georgia

## Abstract

Malignant cardiac tumors are extremely rare, representing only 25% of all cardiac tumors, and angiosarcoma is the most common subtype. Myxofibrosarcomas are one of the rarest forms of cardiac malignant tumors. These tumors can silently grow and produce no or few symptoms until the tumor is large enough to obstruct blood flow. The definitive treatment is negative margin resection, if possible. Most cardiac tumors have a limited response to chemotherapy and radiotherapy. Therefore, surgical treatment is considered the best option. Our patient is a 57-year-old Caucasian postmenopausal female who presented with dyspnea, shortness of breath, and fatigue. Echocardiography confirmed the presence of a mass in the left atrium. A median sternotomy was performed with aortic and bicaval cannulation. Right atriotomy was performed, and the left atrium was exposed through the transseptal approach. A rounded smooth-surfaced mass was found in the left atrium that was 3.5 × 4.5 cm in size. The mass had a prominent and firm attachment point with a wide-based stalk in the pulmonary vein-right atrium border area. The tumor was completely excised, and the excision point was strengthened with a running suture. The following pathologic diagnosis was finally made: myxofibrosarcoma, FNCLCC (National Federation of Cancer Centres) Grade 2. Immunohistochemistry showed positivity for Epithelial membrane antigen (*EMA*), desmin, calretinin, Ki67, Smooth Muscle Actin (*SMA*), and S100. Given the rarity of cardiac malignant tumors, we thought preoperatively that this particular tumor could be a myxoma although it did not have the classical attachment point with a stalk at the interatrial septum. Our case is an example of how fast a cardiac sarcoma can grow. Nine months before the presentation, the patient underwent an echocardiography that did not show any signs of tumor growth. The estimated time of growth was 9 months or less. We followed our patient, performing a computer tomography scan and echocardiography 1 month after surgery, and these did not show any signs of tumor growth.

## 1. Introduction

Malignant cardiac tumors are extremely rare, representing only 25% of all cardiac tumors, and angiosarcoma is the most common subtype [1, 2]. Myxofibrosarcomas are one of the rarest forms of cardiac malignant tumors. These tumors of mesenchymal origin can be found in different locations in the heart—the atria, ventricles, and blood vessels such as the aorta, pulmonary artery, and pulmonary veins. These tumors can silently grow and produce no or few symptoms until the tumor is large enough to obstruct blood flow [3]. Symptoms caused by an obstruction are dyspnea, chest pain, and congestive heart failure. The definitive treatment is a negative margin resection, if possible. In our case, negative margin resection was not confirmed during the operation. Unfortunately, the tumors are not always resectable, and this depends on the extent and depth of the tumor invasion. Most cardiac tumors have a limited response to chemotherapy and radiotherapy. Therefore, surgical treatment is considered the best option [4]. Chemotherapy and radiation therapy can be used to debulk the tumor before resection or it can be used in a positive margin resection when total excision is not possible. In this particular case, we did not use any adjuvant therapy because it was unknown if the tumor is malignant, and there was no indication for that. Overall, this kind of tumor has a very poor prognosis for multiple reasons, including extensive invasion of the tumor into nearby structures and metastasis at the time of diagnosis [5].

## 2. Case Presentation

**Figure 1:**
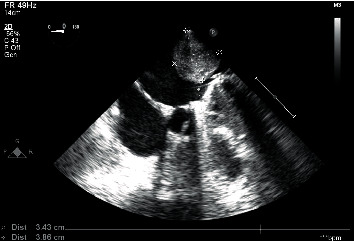
Echocardiography showing a mass in the atrium before surgery.

**Figure 2:**
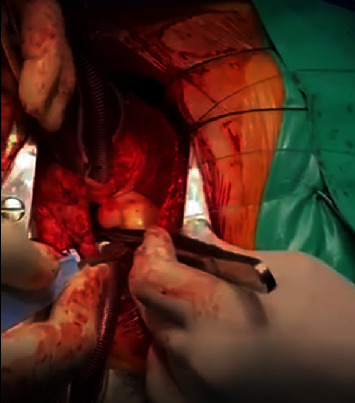
Atriotomy and the transseptal approach exposing the tumor.

**Figure 3:**
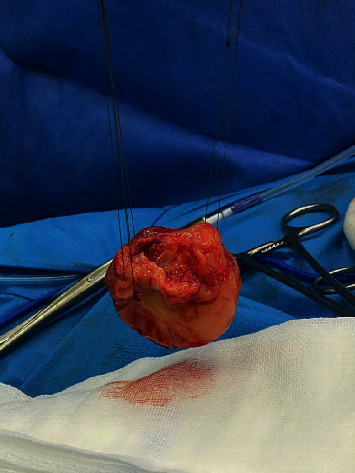
Cardiac sarcoma after excision.

**Figure 4:**
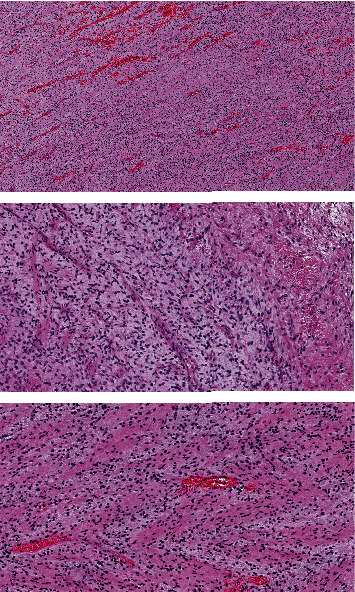
Histology of Myxofibrosarcoma, FNCLCC Grade 2.

Our patient is a 57-year-old Caucasian postmenopausal female who presented with dyspnea, shortness of breath, and fatigue. Echocardiography was performed that showed a mass in the left atrium which was presumably a myxoma or a thrombus. The patient was then transferred to a more specialized hospital in the capital city. After transportation, the patient was admitted to the hospital with the same symptoms of shortness of breath and fatigue. Echocardiography confirmed the presence of a mass in the left atrium (Figure 1). ECG did not reveal any abnormalities. The patient's condition started to deteriorate. Hemoptysis and nasal bleeding were present. The patient's extremities were cold with a capillary refill of >3 sec, and the jugular veins were distended. The patient was sitting in bed because of shortness of breath. A diagnosis of bilateral pneumonia was made. Vital signs at that moment were heart rate, 125 beats per minute without any signs of arrhythmia; blood pressure, 120/80 mmHg; respiratory rate, 32–28 BMP; and SPO2, 93–94% on an O2 cannula of 15 L/min; and bilateral crackles were noted on chest auscultation. The patient's condition started to deteriorate quickly, and it was decided to intubate the patient and start mechanical ventilation because of progressive and rapid respiratory distress that involved extra muscle groups for respiration. Vital signs before intubation and mechanical ventilation were respiratory rate 44 BMP and SPO2, 86–87%. Before intubation echocardiography was performed again, it showed a mass causing blood flow obstruction at the level of the mitral valve, which resulted in blood stasis in the pulmonary system and led to pulmonary edema (mean pressure gradient across the mitral valve, 27 mmHg). It was decided to operate immediately and remove the tumor as fast as possible because cardiogenic shock had developed. Additionally, the patient had a previous mitral valve replacement because of mitral regurgitation and a mechanical valve and tricuspid valve annuloplasty because of tricuspid regurgitation 9 months before this presentation. Echocardiography 9 months before did not show any abnormalities. A median sternotomy was performed with aortic and bicaval cannulation. Right atriotomy was performed, and the left atrium was exposed through the transseptal approach. A rounded smooth-surfaced mass was found in the left atrium that was 3.5 × 4.5 cm in size (Figure 2). The mass had a prominent and firm attachment point with a wide-based stalk in the pulmonary vein-right atrium border area. The mass had a light appearance with signs of vascularization, and it almost filled the right atrium. The tumor was completely excised (Figure 3), and the excision point was strengthened with a running suture. The following pathologic diagnosis was finally made: myxofibrosarcoma (Figure 4), FNCLCC Grade 2. Immunohistochemistry showed positivity for Epithelial membrane antigen (*EMA*), desmin, calretinin, Ki67, Smooth Muscle Actin (*SMA*), and S100. We followed our patient, performing a computer tomography scan and echocardiography 1 month after surgery, and these did not show any signs of tumor growth.

## 3. Discussion

The growth pattern is different in cardiac sarcomas and cardiac myxomas. Given the rarity of cardiac malignant tumors, we thought preoperatively that this particular tumor could be a myxoma although it did not have the classical attachment point with a stalk at the interatrial septum. The tumor did not show the pattern of invasion into the myocardium or other surrounding tissues, and it was freely moving and obstructing the blood flow through the mitral valve during systole. Our case is an example of how fast a cardiac sarcoma can grow. Nine months before presentation, the patient underwent an echocardiography that did not show any signs of tumor growth. The estimated time of growth was 9 months or less. This type of cardiac sarcoma is more amenable to surgical treatment with the goal of a negative margin resection because the attachment point is clearly identifiable and there was no invasion into the surrounding tissues. This leads to better surgical outcomes and better cardiac function as long as there is no need for cardiac reconstruction and reduction of volume in the ventricles or atria because of the myocardial tumor invasion. It is extremely important to have malignant tumors such as sarcomas on the differential when dealing with heart tumors because early diagnosis and negative margin resection yield a better chance of survival and lead to faster rehabilitation.

## Data Availability

The [patient history, radiology and other imaging, ECG, histology and morphology, and other types of data] data used to support the findings of this study are available from the corresponding author upon request.

## References

[1] Ramlawi B., Leja M. J., Abu Saleh W. K. (2016). Surgical Treatment of Primary Cardiac Sarcomas: Review of a Single-Institution Experience. *The Annals of Thoracic Surgery*.

[2] Randhawa J. S., Budd G. T., Randhawa M. (2016). Primary Cardiac Sarcoma: 25-Year Cleveland Clinic Experience. *American Journal of Clinical Oncology*.

[3] Putnam J. B., Sweeney M. S., Colon R., Lanza L. A., Frazier O. H., Cooley D. A. (1991). Primary Cardiac Sarcomas. *The Annals of Thoracic Surgery*.

[4] Shanda H., Blackmon M. D., Michael J., Reardon M. D. (2009). Surgical Treatment of Primary Cardiac Sarcomas. *Texas Heart Institute Journal*.

[5] Bakaeen F. G., Reardon M. J., Coselli J. S. (2003). Surgical outcome in 85 patients with primary cardiac tumors. *American Journal of Surgery*.

